# Anthropomorphic Breast and Head Phantoms for Microwave Imaging

**DOI:** 10.3390/diagnostics8040085

**Published:** 2018-12-18

**Authors:** Nadine Joachimowicz, Bernard Duchêne, Christophe Conessa, Olivier Meyer

**Affiliations:** 1 Group of Electrical Engineering, Paris (GeePs: CNRS—CentraleSupélec—Université Paris-Sud—Sorbonne Université), 91190 Gif-sur-Yvette, France; christophe.conessa@unicaen.fr (C.C.); olivier.meyer@geeps.centralesupelec.fr (O.M.); 2 Laboratoire des Signaux et Systèmes (L2S, UMR 8506: CNRS—CentraleSupélec—Université Paris-Sud), 91190 Gif-sur-Yvette, France; bernard.duchene@l2s.centralesupelec.fr

**Keywords:** microwave imaging, breast cancer detection, brain stroke monitoring, dielectric characterization, UWB breast and head phantoms

## Abstract

This paper deals with breast and head phantoms fabricated from 3D-printed structures and liquid mixtures whose complex permittivities are close to that of the biological tissues within a large frequency band. The goal is to enable an easy and safe manufacturing of stable-in-time detailed anthropomorphic phantoms dedicated to the test of microwave imaging systems to assess the performances of the latter in realistic configurations before a possible clinical application to breast cancer imaging or brain stroke monitoring. The structure of the breast phantom has already been used by several laboratories to test their measurement systems in the framework of the COST (European Cooperation in Science and Technology) Action TD1301-MiMed. As for the tissue mimicking liquid mixtures, they are based upon Triton X-100 and salted water. It has been proven that such mixtures can dielectrically mimic the various breast tissues. It is shown herein that they can also accurately mimic most of the head tissues and that, given a binary fluid mixture model, the respective concentrations of the various constituents needed to mimic a particular tissue can be predetermined by means of a standard minimization method.

## 1. Introduction

Due their non-ionizing nature and to the low cost and portability of the equipment, microwaves arouse a keen interest for biomedical applications. Furthermore, several studies have shown that, at these frequencies, the various human biological tissues show significant differences in their dielectric properties [1]. This is the reason why, at the present time, a lot of work is devoted to biomedical microwave imaging, more specifically for breast cancer detection and brain stroke monitoring. It can be noted that in these last two applications, the interest of microwave imaging lies in the dielectric contrast which may exist between normal healthy tissues and malignant [2] or stroke-affected [3] ones and, in turn, the magnitude of this contrast depends upon the nature of the disease, i.e., ischemic or hemorrhagic for the stroke and located in fat or in fibroconnective-glandular tissues for the breast tumor. For the latter case, contrasts as high as 10:1 are reported in Reference [2] between malignant and healthy adipose breast tissues; however, those that can be found between tumors and normal fibroconnective–glandular tissues are less than 10%, which renders the detection of such tumors with microwave imaging challenging.

Although microwave imaging is still an emerging technique that is not yet recognized as an alternative to magnetic resonance imaging (MRI) or X-ray computerized tomography (CT), several microwave imaging systems dedicated to breast tumor detection [4,5,6,7,8] (see Reference [9] for a comprehensive comparison of the various systems that concern this application) and brain stroke monitoring [10,11,12,13] are already at the clinical trial level.

However, before such a trial, the imaging systems need to be tested on reference anthropomorphic phantoms in order to assess and compare their performances in controlled realistic configurations. These reference phantoms should satisfy several requirements: Particularly, their structure must be close to that of the targeted human body part (breast or head), the dielectric properties of their constitutive materials must be close to that of the various biological tissues of the abovementioned part, and finally, their shape and dielectric properties must be stable over time in order that the phantom can be used as a benchmark.

One of the main difficulties encountered when looking for a tissue mimicking material (TMM) is the large dispersivity of soft tissue dielectric properties in the microwave frequency range. Thus, a lot of mixtures have been considered as TMMs [14], among which jelly mixtures based upon oil-in-gelatin dispersions [15,16,17,18] or upon water–agar or water–gelatin blends [19] and gel substances based upon water–polythene powder-TX-151 mixtures [20] are certainly among the most promising materials, as, in addition to accurately simulating the dispersive dielectric properties of the various human tissues in a large frequency range, they are relatively easy to produce and their mechanical properties allow the construction of anthropomorphic phantoms. Hence, the abovementioned mixtures fulfill the first two requirements outlined in the previous paragraph; however, they fail in satisfying the last one. Indeed, the dielectric and mechanical properties of phantoms based upon these TMMs are unstable over time. This is due either to evaporation or diffusion phenomena between layers of different gelatin concentrations [15] for the water–gelatin-based mixtures or to interaction with air if they are not very carefully shielded from the environment for the oil-in-gelatin dispersions [21,22]. Furthermore, with these materials, it is not always easy to avoid air bubbles getting trapped in the mixtures without specific equipments. If such bubbles are present, they would behave as small high-contrasted diffractors, which would greatly perturb the electromagnetic field within the phantom. Solid TMMs do not present these drawbacks; however, phantoms made of such materials [23] are not reconfigurable as solid TMMs are not adjustable in order to account for changes linked, for example, to the appearance of a tumor or of a stroke. By contrast, liquid mixtures allow us to avoid air bubbles and stability problems and they are adjustable, as they can easily be replaced.

Fluid TMMs based upon mixtures of Triton X-100 (TX-100, a non-ionic surfactant) and water have already been used to mimic the various breast tissues [24,25]; however, they cannot account for the high conductivity of many tissues at high frequencies. We have shown that adding salt to these mixtures allows us to get both permittivity and conductivity close to that of the various breast tissues over the 0.5–6 GHz range [22]. It is shown herein that, in fact, these mixtures are also good TMMs for head tissues. Furthermore, the respective concentrations of the various constituents needed to mimic a given tissue can be approximately deduced from a binary fluid mixture model involving TX-100 and salted water. In Reference [22], the dielectric properties of such mixtures were also shown to be stable over time periods as long as 1 year. Such a time stability is obtained by taking the precaution of extracting the TMMs from the phantom rigid structure required to contain and separate the TMMs that correspond to the different tissues, and to keep them away from light in sealed containers to avoid evaporation.

Concerning the phantom’s rigid structure, recent progress in additive manufacturing now allows us to build up relatively easily reproducible 3D-printed complex structures from STL (stereolithography) files that describe their surfaces. For anthropomorphic structures, these STL files can be obtained from MRI or X-ray CT scans. Finally, one further advantage of 3D-printed phantoms is that the STL file can also be used to perform numerical simulations along with experimental validations. Before the design of the breast and head phantoms presented herein, other phantoms had already been built up in this way [25,26,27,28]; however, their structures were not suitable to be filled up with several fluid TMMs, as one is made of a unique cavity while the others are made of several parts which are intended to be used as temporary molds where gel-based breast or head parts are formed. The novelty, herein, was that the phantoms comprise several cavities intended to be filled up with different fluid TMMs. Since then, similar breast phantoms have been proposed [29,30,31].

## 2. The Phantoms

### 2.1. 3D-Printed Structures

**Figure 1:**
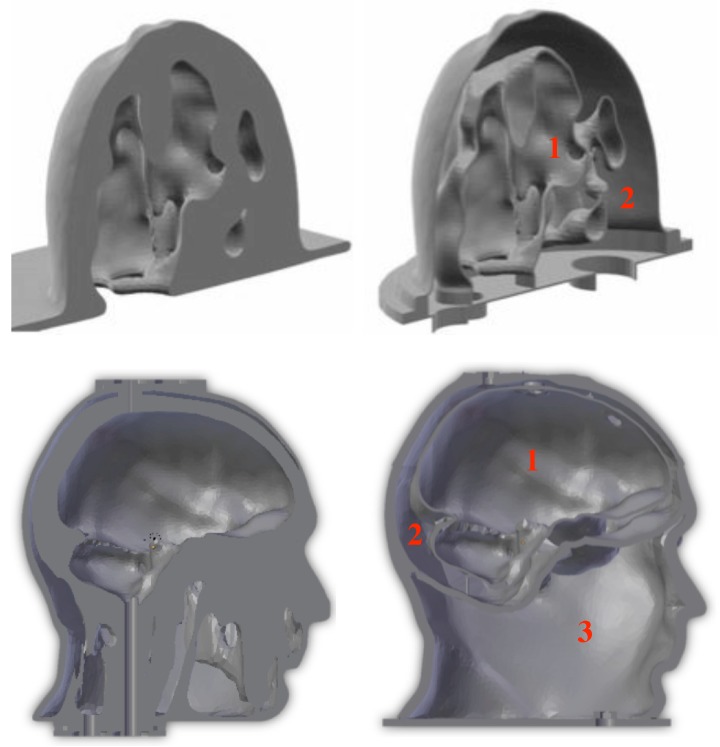
Sagittal sections of the breast (**up**) and head (**down**) phantoms derived from the original STL (stereolithography) files (**left**) and from the modified ones (**right**). The red numbers indicate the various cavities that contain the different TMMs corresponding to: (1) fibroglandular or heterogeneous mix tissues, (2) fatty tissues (**up-right**), and (1) brain, (2) cerebrospinal fluid, (3) miscellaneous tissues (**down-right**).

**Figure 2:**
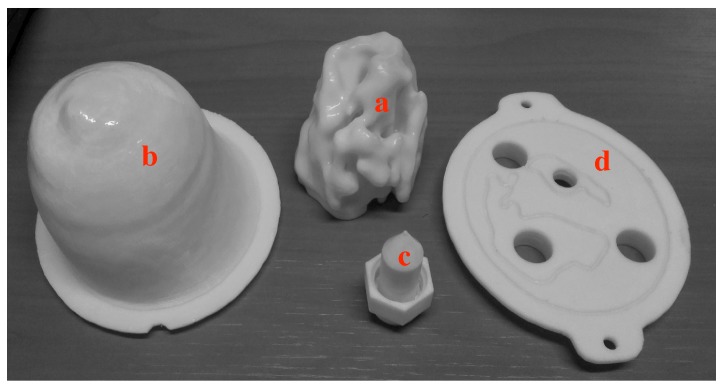
The different parts of the GeePs-L2S breast phantom: (**a**) The inner part contains the fibroglandular or heterogeneous mix tissue mimicking material (TMM); (**b**) the outer shell contains the fatty TMM; (**c**) the removable inclusion contains the tumor-like TMM; and (**d**) the support plate holds the different parts in place.

**Figure 3:**
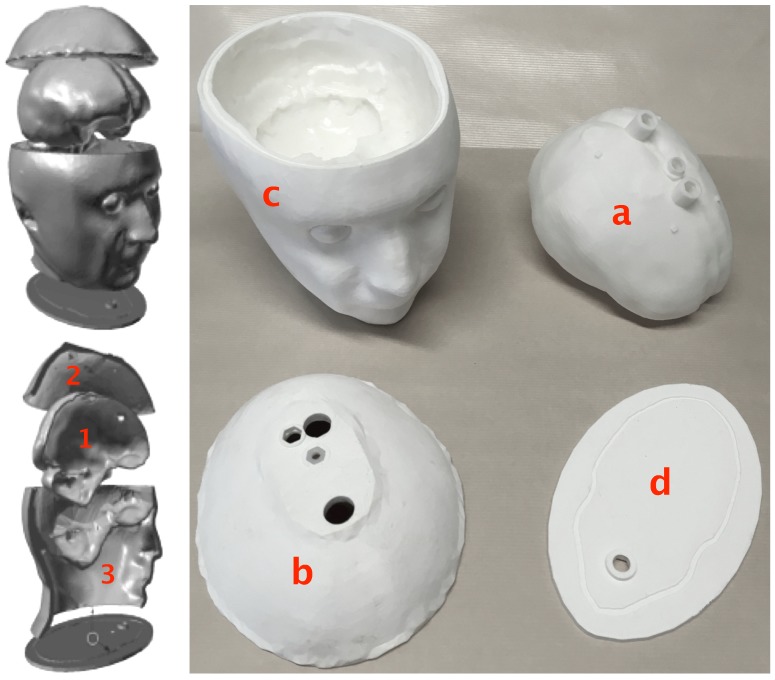
The different parts of the head phantom: (1) The inner tank (**a**) contains the brain TMM, (2) the upper cavity contains the cerebrospinal fluid (CSF) TMM, and (3) the lower one contains an average tissue medium mimicking mixture. The top and bottom of part (**c**) are clipped, respectively, to the part (**b**) and to the plate (**d**) by means of a tenon–mortise system that runs all around the joints, and the different parts are glued once in place, while the brain tank is held in place by several stops.

Both breast and head phantoms are produced in the same way. Their structures are made of acrylonitrile butadiene styrene (ABS) and built up by additive manufacturing from STL files obtained by modifying original files available in the literature that describe anatomically realistic breast and head structures derived from MRI scans. Hence, the original file corresponding to the breast phantom comes from the University of Wisconsin–Madison [25], while that corresponding to the head phantom comes from the Athinoula A. Martinos Center for Biomedical Imaging at Massachusetts General Hospital [32]. These files have been modified by means of a computer-aided design software so as to separate three distinct cavities. This results from a trade-off between the preservation of highly dielectrically contrasted regions around the area of interest (i.e., the brain for the head phantom) and the minimization of the number of ABS internal walls that raise leakage and field perturbation issues. The phantoms are printed in several parts that are clipped and glued together and the seals are weatherproofed. Figure 1 displays sagittal sections of the breast and head phantoms produced from the original and modified STL files, while Figure 2 and Figure 3 display exploded views that show the different parts of the latter, respectively.

Concerning the breast structure, it is denoted as the GeePs-L2S (or Supelec) breast phantom. It has already been used as a reference phantom in the framework of Cost Action TD1301 MiMed (http://cost-action-td1301.org) and several publications report experimental results collected with this phantom by means of various microwave imaging systems [33,34,35,36].

In this phantom, cavities 2 and 1 (Figure 1—top right) correspond to a typical distribution of fatty and fibroglandular or heterogeneous mix tissues, respectively, while the third one (Figure 2c) can be placed at different locations in order to account for the presence of a tumor.

As for the head structure, it includes three fixed cavities. Cavities 1 and 2 (Figure 1—down right) are filled up from the top with brain and cerebrospinal fluid (CSF) TMMs, respectively, and cavity 3 can be filled from the bottom of the structure with mixtures whose dielectric properties can be adjusted in order to fit those of various tissues, such as bone, muscle, blood or a medium whose properties are an average of that of these tissues. Of course, the latter cavity must filled up before the former ones with the head upside down and the filling hole must be tightly closed before turning the phantom right side up. It can be noted that during this operation, it is difficult to avoid a little bit of air remaining in the cavity; however, once the phantom is right side up, this air will rise to the level of the nasal cavity where it is naturally present in a real human head.

Except for the outer shell of the head, which is relatively thick (≈8 mm, i.e., the thickness of the skull) in order to get a good rigidity, for both phantoms, the thickness of the ABS structures is 1.5 mm. This results from a trade-off between wall stiffness, structure tightness, and low field perturbation. Indeed, at a frequency of 2.45 GHz, the values of the ABS dielectric parameters are $\epsilon_{r}=3$ and $\sigma =4\times 10^{-3}$ S/m, which is far from the dielectric properties of the various biological tissues and, hence, leads us to opt for a thin structure in order to minimize the perturbation of the field inside the phantoms. However, this trade-off is not satisfactory. Indeed, on one hand, a 1.5-mm thickness is not sufficient to ensure a perfect waterproofing of the phantom, but leakages can be avoided by smoothing the structure by means of acetone vapor and by coating it with epoxy resin. On the other hand, despite their thinness, it has been experimentally [37] and numerically [38] shown that due to the high dielectric contrast with respect to the various biological tissues, the ABS walls perturb the field significantly. Concerning the breast structure, a solution proposed in Reference [29] consists of using conductive ABS whose dielectric parameters (i.e., $\epsilon_{r}\approx 10$ and σ≈0.4 S/m at 2.45 GHz, see [29]) are closer to that of adipose tissues ($\epsilon_{r}\approx 5$ and σ≈0.1 S/m, see Table 1) than the normal one. It can be noted that at 1 GHz, which should be the central frequency of the band considered for brain stroke monitoring, as will be seen later on, the parameters of conductive ABS are also very close to that of the bone ($\epsilon_{r}\approx 12$ and σ≈0.2 S/m, see Table 2 and Reference [29]); hence, this material is appropriate for the outer shell of the head that represents the skull and it could be used to print parts “b” and “c” (see Figure 3) of future versions of the head phantom. However, this material is not adequate for the inner walls of the phantom and a printable material whose parameters are close to that of the brain is still to be found. Finally, although this has not been done therein, the phantoms can be improved by plastering their external shell with flexible skin mimicking mixtures based upon graphite, carbon black, and silicone rubber [29,39] or urethane [40], that, in addition, could also solve the problems of leakage through the external wall.

### 2.2. Tissue Mimicking Mixtures

As underlined above, in addition to being good TMMs for breast tissues, liquid mixtures made of TX-100 and salted water can mimic almost all the head tissues over a large frequency band with good precision. Furthermore, given a temperature and a frequency band, the concentrations of TX-100 and salt in the mixture needed to mimic a specific tissue can be approximately predetermined with a binary mixture model, such as the Böttcher’s one [41] that yields $\epsilon_{m}$, the complex permittivity of the TMM, as a function of ($\epsilon_{1}$, $V_{1}$) and ($\epsilon_{2}$, $V_{2}$), the permittivities and volume fractions of TX-100 and salted water, respectively. By accounting for the fact that $V_{1}+V_{2}=1$, $\epsilon_{m}$ can be expressed without $V_{2}$:

(1) $$\epsilon_{m}=\epsilon_{2}+\left[3\;V_{1}\;\epsilon_{m}\left(\epsilon_{1}-\epsilon_{2}\right)/\left(2\epsilon_{m}+\epsilon_{1}\right)\right].$$

Elsewhere, Debye [42] and Cole–Cole [43,44] models have been developed for most of the human body tissues to describe the behavior of their complex permittivities $\epsilon_{t}$ as functions of the frequency. Particularly, in Reference [42], an accurate Debye model can be found to describe the permittivity of breast tissues with adipose tissue content in the range 85–100%, defined as group 3 in Reference [2], and it has been shown in Reference [22] that, in the 0.5–6 GHz frequency band, the complex permittivity of this tissue group is very close to that of TX-100, so that we have a model for $\epsilon_{1}$:

(2) $$\epsilon_{1}\left(\omega \right)=3.14+1.6/\left(1+j\;13.56\times 10^{-12}\;\omega \right)+0.036/\left(j\;\omega \epsilon_{0}\right),$$

where ω is the angular frequency, *j* is the imaginary unit, and $\epsilon_{0}$ the dielectric permittivity of vacuum. It has been shown that the permittivity of TX-100 varies only very slightly with the temperature in the range 15–37 °C. Concerning the salted water, a parametric model can be found in Reference [45] that expresses $\epsilon_{2}$ as a function of the frequency, the salinity, and the temperature.

Hence, the mixture component concentrations needed to mimic a specific tissue can be determined by fitting the mixture model $\epsilon_{m}$ to the permittivity of the tissue $\epsilon_{t}$ at several discrete frequencies *f* over the frequency range of interest, i.e., by minimizing the following cost functional:

(3) $$J=\sum_{f}\;w_{f}|\epsilon_{m}-\epsilon_{t}{|}_{f}^{2},$$

where $w_{f}=1/|\epsilon_{t}{|}_{f}^{2}$.

This can be done in an iterative way by means of a Gauss–Newton method [46]. The solution $x={\left(V_{1},S_{m}\right)}^{†}$ (where ${}^{†}$ indicates the transposition, $V_{1}$ the volume fraction of TX-100, and $S_{m}$ the NaCl concentration of the mixture) at iteration step k+1 then reads:

(4) $$x^{k+1}=x^{k}-H^{-1}\left(x^{k}\right)g\left(x^{k}\right).$$

In the above equation, ***g*** and ***H*** are the gradient and the approximate Hessian of *J*, respectively:

(5) $$\begin{array}{c} g=2\sum_{f}\;w_{f}\;ℜe{\left[{\left(\epsilon_{m}-\epsilon_{t}\right)}_{f}^{*}\;\epsilon_{m}^{\prime }\right]}_{f},\\ H=2\sum_{f}\;w_{f}\;ℜe{\left({\epsilon^{\prime }}_{m}^{*}\;{\epsilon^{\prime }}_{m}^{†}\right)}_{f}, \end{array}$$

where $\epsilon_{m}^{\prime }={\left(\partial \epsilon_{m}/\partial V_{1},\partial \epsilon_{m}/\partial S_{m}\right)}^{†}$ and ${}^{*}$ indicates the conjugate. By accounting for Equation (1), $\epsilon_{m}^{\prime }$ becomes:

(6) $$\epsilon_{m}^{\prime }=\left(\begin{array}{c} 3\;\gamma \;(\epsilon_{1}-\epsilon_{2})/\left(4\delta \right)\\ \left[\left(\gamma \;\left(3V_{2}-1\right)+4\epsilon_{1}\right)\;/\;\left(4\;V_{2}\;\delta \right)\right]\;\partial \epsilon_{2}/\partial S_{2} \end{array}\right),$$

with:

$$\begin{array}{lll} \gamma & = & \delta -\eta ,\;\delta ={\left(\eta^{2}+8\;\epsilon_{1}\;\epsilon_{2}\right)}^{1/2}\\ \eta \; & = & \;\epsilon_{1}-2\;\epsilon_{2}-3\;V_{1}\;\left(\epsilon_{1}-\epsilon_{2}\right). \end{array}$$

The term $\partial \epsilon_{2}/\partial S_{2}$ can be straightforwardly deduced from the salted water parametric model. The above described iterative method converges very rapidly towards a stable solution that generally depends very little on the initial guess, which allows us to choose $x^{0}$ in an empirical way. It can be noted that for a given mixture, due to the discrepancy between the dielectric parameter measured values and those given by Böttcher’s model, the TX-100 and salt concentrations must be experimentally refined around the solution given by the latter in order to get closer to the expected permittivity values.

Table 1 recalls the results of Reference [47] concerning the breast TMMs at a temperature of 37 °C and a frequency of 2.45 GHz. It displays the TX-100 and salt concentrations obtained by fitting the Böttcher’s and Debye models over the 0.5–6 GHz range and the measured and expected (given by the Debye model) dielectric properties of the various mixtures. The “measured” values are the means of measurements performed with three different apparatuses dedicated to the characterization of liquid dielectric material properties, several measurements being made with each system. The first one, denoted as S1 in the following, consists of a coaxial waveguide coupled to an Agilent E8364C (Keysight Technologies, Santa Rosa, CA, USA) vector network analyzer (VNA) on one side and, on the other side, to a circular cylindrical cell by means of a dielectric coaxial tight window; this cell is made of a 7-mm-diameter circular waveguide intended to be filled up with the liquid dielectric under test and is ended by a short circuit [48]. The other two systems consist of open-ended coaxial sensors: A Keysight 85070D high-temperature dielectric probe coupled to an HP 8753E VNA (Keysight Technologies, Santa Rosa, CA, USA) and a homemade one connected to a Rodhe & Schwarz ZVB8 VNA (Rodhe & Schwarz France, Meudon-la-forêt, France) and built up from a 3.6-mm-diameter, 15-cm-long, Teflon-filled copper rigid coaxial cable. The uncertainties that appear in Table 1 are the standard deviations of all the measurements performed by means of the three systems and, below 4.5 GHz, these deviations are generally less than 5% of the mean values, except for the conductivity of group G3, as the latter is very low.

The TX-100–salted water mixtures are very easy to produce; however, for TX-100 volume percentages in the range 40–50%, at low temperature and salt concentration, the mixture is rather viscous. It can be noted that very few TMMs are concerned by this problem (among those presented herein, only G2 of Table 1 falls into this category), but, for the latter, the mixture components are warmed separately, then mixed and vigorously stirred, left to rest at 45 °C for a few minutes until air bubbles vanish, and poured into the cavity while it is still warm.

Table 2 displays the results obtained in the same conditions for the head TMMs at 1 GHz. Note that the last two columns display the expected values given by the Cole–Cole models of References [43,44]. In this table, the brain is considered as a blend of white and grey matters (75% of white matter and 25% of grey matter) and “bone” refers to the cortical bone. Here again, the standard deviations are generally less than 5% of the mean values, except for CSF. This exception is linked to system S1, whose measurement results become less accurate as the permittivity increases, due to concomitant lowering of the cutoff frequency in the measuring cell.

**Figure 4:**
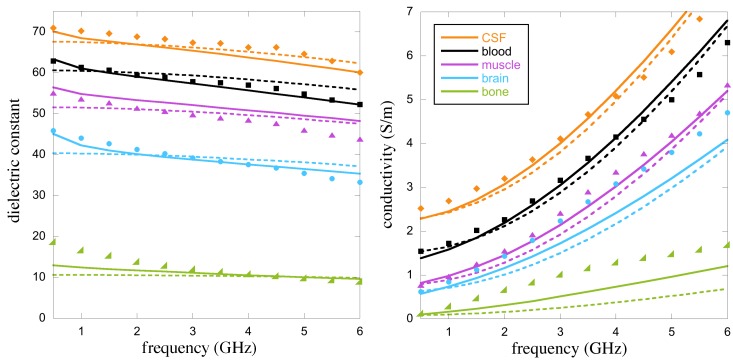
Means of the dielectric properties of various TMMs measured with three different set-ups (markers), compared to the results obtained by means of the tissue Cole–Cole models (full lines) and to those obtained with Böttcher’s binary mixture law (dashed lines).

Figure 4 displays the results obtained over the 0.5–6 GHz band. Measured, predicted (from Böttcher’s model) and expected (from the Cole–Cole model) properties are in good agreement for almost all the tissues except, maybe, the measured values of conductivity for the brain and the bones that deviate a little bit from the expected values. It is worth noting that the variability of human tissue dielectric properties is very important. In the frequency band considered herein, it is evaluated in Reference [1] to be in the range ±Δ% (where 5≤Δ≤10) of the permittivity values given by the Cole–Cole models. Concerning the breast tissues considered in Table 1, the variability is even more important as each group spans tissues with a large heterogeneity in their adipose content (see [2]—Figures 9 and 10). This means that for almost all the TMMs considered herein except the bone mimicking one, the measured values fall within the uncertainty range of the Debye and Cole–Cole models, if the variability ranges of the tissue dielectric properties can be considered as the uncertainty ranges of the parametric models.

Furthermore, better fitting between predicted and expected properties of the various TMMs could be obtained with a narrower operating frequency range, and, although a lower resolution should be expected, the 0.6–1.5 GHz band would be more appropriate for brain stroke monitoring that requires an important penetration depth of the interrogating wave and where the range 1.5–4 GHz is a kind of “forbidden band” due to the strong attenuation of the waves within the head [3,49,50].

## 3. Conclusions

It has been shown herein that reference phantoms can be built up from 3D-printed structures and fluid TX-100–salted water mixtures. Such mixtures can mimic most of the breast and head tissues with a good precision concerning their dielectric properties over a large frequency range, and they are easily adjustable and reproducible. Furthermore, the respective proportions of the different mixture constituents needed to mimic a particular tissue can be approximately predetermined by means of a binary mixture model. Admittedly, these phantoms are very simplified compared to the real human breast or head and are less realistic than some other phantoms that can be found in the literature; however, they have the advantage of being stable over time and easy to produce and, in addition, they preserve the areas of high dielectric contrast that are of interest to the applications considered herein, so that they can be considered as anthropomorphic. Note that they could be refined to be more realistic. Particularly for the head phantom, the lower part of the structure could be redesigned so as to delineate the buccal and nasal cavities, the eyeballs, and the muscles; however, this would need more ABS walls, which means field perturbation and leakage issues, for an improvement that should probably be minimal, as these parts are far from the area targeted by the brain stroke monitoring application. Furthermore, this would contradict the goal of this study that consists of the conception of simple phantoms that anyone involved in the field of microwave imaging could easily produce. The major drawback of these phantoms lies in the limited number of materials that can be used in additive manufacturing, which does not allow us to get rigid structures whose dielectric properties are close to that of some specific human tissues and particularly to that of the brain, but this drawback will certainly be overcome in the near future due to the rapid progress of 3D-printing technology that increasingly allows more and more materials to be processed.

## Figures and Tables

**Table 1:** Composition and properties of breast TMMs at 2.45 GHz and 37 °C (group: T = tumor, G1 = fibroglandular tissue, G2 = heterogeneous mix tissue, G3 = fatty tissue).

Group	Mixture Composition		Averaged Measurements		Debye Model	
	TX-100	NaCl	$\epsilon_{r}$	σ	$\epsilon_{r}$	σ
	(vol %)	(g/L)		(S/m)		(S/m)
T	18	4.0	56 ± 2	1.79 ± 0.06	53	1.8
G1	28	3.5	47 ± 1	1.61 ± 0.08	46	1.6
G2	41	0	37.8 ± 0.3	1.12 ± 0.05	37	1.1
G3	100	0	4.76 ± 0.04	0.18 ± 0.03	5	0.1

**Table 2:** Composition and properties of head TMMs at 1 GHz and 37 °C versus the values inferred from Cole–Cole models.

Tissue	Mixture Composition		Averaged Measurements		Cole-Cole	
	TX-100	NaCl	$\epsilon_{r}$	σ	$\epsilon_{r}$	σ
	(vol %)	(g/L)		(S/m)		(S/m)
Brain	38	5.2	44 ± 2	0.84 ± 0.03	42	0.7
CSF	6	13.7	70 ± 7	2.7 ± 0.2	68	2.5
Muscle	24	5.0	54 ± 2	0.97 ± 0.03	55	1.0
Bone	75	0.8	16.7 ± 0.8	0.30 ± 0.04	12	0.2
Blood	14	9.4	61 ± 3	1.72 ± 0.07	61	1.6
